# Phase Separation: Direct and Indirect Driving Force for High-Order Chromatin Organization

**DOI:** 10.3390/genes14020499

**Published:** 2023-02-15

**Authors:** Xiaoli Li, Ziyang An, Wenqing Zhang, Feifei Li

**Affiliations:** 1 Division of Cell, Developmental and Integrative Biology, School of Medicine, South China University of Technology, Guangzhou 510006, China; 2 Department of Cell Biology and Genetics, Core Facility of Developmental Biology, Chongqing Medical University, Chongqing 400016, China; 3 State Key Laboratory of Biocontrol, School of Life Sciences, Sun Yat-Sen University, Guangzhou 510275, China

**Keywords:** phase separation, 3D chromatin organization, chromatin functional compartmentalization, transcription regulation

## Abstract

The multi-level spatial chromatin organization in the nucleus is closely related to chromatin activity. The mechanism of chromatin organization and remodeling attract much attention. Phase separation describes the biomolecular condensation which is the basis for membraneless compartments in cells. Recent research shows that phase separation is a key aspect to drive high-order chromatin structure and remodeling. In addition, chromatin functional compartmentalization in the nucleus which is formed by phase separation also plays an important role in overall chromatin structure. In this review, we summarized the latest work about the role of phase separation in spatial chromatin organization, focusing on direct and indirect effects of phase separation on 3D chromatin organization and its impact on transcription regulation.

## 1. Introduction

The site for storing genetic information in eukaryotes is the nucleus, and chromatin is accommodated in its limited space through multi-level organization and compaction. Three-dimensional chromatin structure is highly correlated with multiple processes, including correct gene expression, DNA replication and so on. In order to gain deep insights into 3D chromatin organization, two kinds of methods have emerged. One is based on the microscope approach, and the other is based on high-throughput sequencing. These methods deepened our understanding of the 3D genome, and proved that chromatin organization is highly degree ordered and unevenly distributed in space [1,2]. The genome enriches RNA factors and proteins into chromatin subcompartments with a diameter of about 0.1–1 µm to complete self-organizations [3,4]. An important example is that the transcription-activated euchromatin and the inactivated heterochromatin are clearly divided into different regions in the eukaryotic nucleus [5]. In addition to genetic material, proteins are also not evenly distributed in the nucleus. It is well known that specific proteins are concentrated in nuclear bodies, such as nucleolus and nuclear speckles [6,7]. These higher concentrations of enzymes and cofactors facilitate complex biochemical reactions.

The mechanism of high-order chromatin organization received much attention in recent years. A few models were proposed and validated to contribute to chromatin architecture, such as self-organization and loop extrusion [3]. More and more evidence showed that liquid–liquid phase separation (LLPS) plays an important role in the 3D genome organization and remodeling [8]. Phase separation refers to the process in which a mixture of components in a solution reaches its lowest free energy by separating into distinct phases, such as a concentrated phase and a diluted phase [9]. It was used to explain the formation of membraneless organelles and bioreactors in cells. With the rapid development of the field, a variety of cellular processes were found to be associated with phase separation within cells, such as cell division, DNA damage repair and X chromosome inactivation [10,11,12]. Phase separation was also proposed to be related to the establishment and reformation of chromatin spatial organization. According to properties of the separated phase and properties of factors that mediate interaction, different mechanisms of phase separation can participate in genome organization, such as liquid–liquid phase separation (LLPS), polymer–polymer phase separation (PPPS), liquid–gel phase separation (LGPS) and liquid–solid phase separation (LSPS) [13]. As recent studies focused mainly on LLPS as the driving force for chromatin structure formation, the phase separation in this review mainly refers to LLPS unless stated otherwise. LLPS also affects chromatin distribution in nucleus by constraining its interactions with nuclear bodies which are also formed by LLPS. Through these direct and indirect influences, phase separation plays key roles in overall chromatin structure and gene expression regulation.

The continuous development of new technologies has provided us with unprecedented insight into the role of LLPS in 3D genome organization. This review first concisely introduces the emergence and development of LLPS, then briefly recounts 3D chromatin organization research. We emphatically summarize the role of LLPS in the formation of 3D chromatin organization at different scales, and discuss the contribution of several important nuclear bodies in the nucleus to the formation of chromatin structure. This can help us better understand the contribution of phase separation to chromatin remodeling and gene transcription regulation, and provide guidance for future studies.

## 2. Snapshot of Liquid–Liquid Phase Separation

As early as 2009, Tony Hyman put forward the concept of LLPS. By studying the nuclear pore complexes (NPCs) and discovering the liquid-like properties of P granules in the reproductive cells of Caenorhabditis elegans, they found that P granules were membrane-free agglomerates formed by phase separation of protein and RNA [14]. Similar to P granules, there are many kinds of liquid droplets in cells [15], including nucleolus, Cajal bodies, promyelocytic leukemia (PML) bodies [16], stress granules (SGs) [17], histone locus bodies [18] and membrane clusters [19]. All these structures lack classical membranes but have high concentrations of specific molecular components and can concentrate enzymatic reactions in a specific cellular location with high efficiency. Many of these membrane-free subcellular structures are formed and maintained by LLPS. In cells, multivalent molecular interactions between proteins and/or nuclei acids can lead to LLPS [8]. Proteins driving phase separation often contain multiple modular domains, and they can combine with each other or other molecules to form biological molecule condensate, which makes phase separation occur. Proteins with intrinsically disordered regions (IDRs) are another important type of multivalent molecule [15]. IDR regions are also called low-complexity domains, which often contain repetitive amino acid sequences and are rich in glycine, serine, glutamine, asparagine, phenylalanine and tyrosine. IDR regions are highly flexible and prone to establishing homogeneous or heterogeneous interactions to induce phase separation [20]. In addition, RNA and DNA also participate in the formation of phase separation [21,22]. 

Biomolecular condensation formed by LLPS has some specific liquid-like characteristics. Typically, it is spherical and can separate or fuse under the shear force to form a smaller or bigger spherical droplet. Fluorescence recovery after photobleaching analysis demonstrated that the molecular exchange between phased droplets and the surrounding environment is fast and frequent. In addition, LLPS is highly dynamic. LLPS can be affected not only by the characteristics of its components, such as electronic characteristics, quantity, reactivity and spatial arrangement, but also by environmental factors, such as temperature, PH and ion concentration [23]. LLPS has been found to play roles in diverse biological processes such as signal transduction, cell cycle, cytokinesis and maintenance of cellular homeostasis [24]. Recent studies showed the attractive influence of LLPS on nuclear organization and transcription regulation across the eukaryotic cells.

## 3. Current View of Spatial Chromatin Organization

The basic structural unit of eukaryotic chromatin is the nucleosome, which is composed of 147 bp of DNA wrapped around a histone octamer and forms a beads-on-a-string structure. Nucleosomes need to be further folded within a micro size nucleus and form higher order chromatin organization [25]. At present, the understanding of spatial genome organization mostly depends on the results of a series of sequencing technologies. For example, Hi-C, ChIA-PET, HiChIP, SPRITE, GAM and so on [26,27,28,29,30]. Using these and image-based techniques, we learned that interphase chromosomes are folded into multiple layers of hierarchical structures. The chromatin can be separated into two compartments named compartment A and B, which correspond to the active and repressed chromatin domain, respectively [26]. At higher resolution, the chromatin is folded into the so-called “topological association domain” (TAD), which tends to be stable across different cell types and highly conserved across species [31]. Within TADs, chromatin loops are formed which correspond to loci interacting more frequently than neighboring sites [32]. 

High-order chromatin organization is closely related to all chromatin-based activity in the nucleus, including transcription, DNA replication and so on. Many studies focus on the mechanism of chromatin organization and remodeling. Although there is evidence that the formation of TAD and loops mainly depends on the loop extrusion model [33,34], the formation of compartments seems to apply a different mechanism. Deletion of main structural proteins showed that although TAD and loops were globally lost, compartments are unaffected and even reinforced [35]. It is worth noting that many proteins related to chromatin structure in the nucleus possess IDRs, and can form liquid condensates under specific conditions, which may underlie the formation of a self-interacting domain in the Hi-C diagram [36,37,38]. Compartments are proposed to be formed by phase separation, and there are a few wonderful reviews describing this [10,38,39]. Recently, theoretical models and experimental studies illustrate that LLPS also contributes to more finer chromatin structure, such as TADs, loops and nucleosomes condensation. These results suggest that phase separation acts as a driving force for high-order chromatin organization and reprogramming. In the next two parts, we comprehensively summarize the direct driving role of LLPS to all layers of high-order chromatin structure, and the indirect impact of LLPS on genome organization by formation of nuclear bodies.

## 4. Phase Separation Directly Impacts All Layers of High-Order Chromatin Organization

### 4.1. Role of LLPS in Nucleosome-Mediated Chromatin Condensation

**Figure 1:**
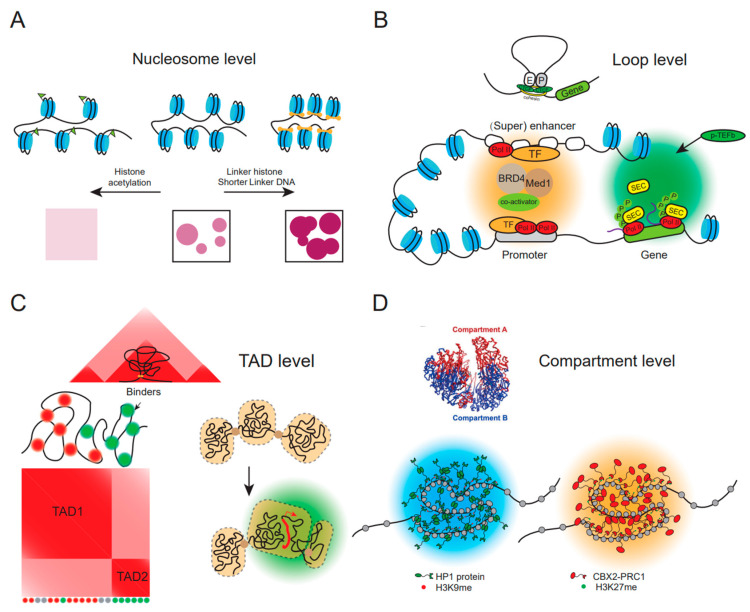
Phase separation contributes to chromatin organization at different levels. (**A**) At nucleosome level, nucleosomes intrinsically condensate chromatin into globules. Linker DNA length, histone H1 and histone modification can dissolve or alter the property of separated phase. (**B**) At chromatin loop level, phase-separated transcriptional condensates are established composing of transcription factors, mediator, co-activator and Pol II, which are important for gene expression. Phosphorylation of the Pol II C-terminal domain drives an exchange from condensates that are involved in transcription initiation to those that are involved in RNA processing. (**C**) At TAD level, TADs can be recreated in computational polymer models by assuming intra-polymer self-interactions. In addition, phase separation controls TAD reorganization to promote specific gene expression and cell fate transitions. (**D**) At compartment level, phase separation contributes to formation of both constitutive and facultative heterochromatin. For constitutive heterochromatin, HP1 recognize and bind H3K9me to trigger the formation of separated aggregates. For facultative heterochromatin, phase separation is driven by CBX2-PRC1 and H3K27me.

In physiologic salt concentrations, 10 nm nucleosomes have the intrinsic ability to condensate chromatin into globules through LLPS (Figure 1A) [38]. Both linker DNA and attractive nucleosome–nucleosome interactions influence the chromatin condensation. Shorter linker length with 10n + 5-bp (e.g., 15, 25, 35) linker pattern favor chromatin LLPS [11,38]. Core histone tails are essential and chromatin cannot condensate without histone tails [40]. Histone tails may mediate phase separation through their interaction with linker DNA. Previous studies also showed that when H2A, H2B, H3 and H4 tail domains are deleted individually, the oligomerization of nucleosomes are impaired [41]. In addition, H2A was shown to form droplets with DNA in vitro, which may also contribute to chromatin condensation [42]. Moreover, linker histone H1 can also phase separate with DNA in living cells with its C-terminal IDR tail and addition of H1 significantly promotes the chromatin phase separation [37]. In agreement with the role of LLPS in mediating nucleosome condensation, disruption of LLPS by 1,6-hexanediol leads to decondensation of nucleosome clutches and more uniform distribution in the nuclei [43]. 

Histone tails emanate from nucleosome core particles and can be modified by different kinds of post-translational modifications, including methylation, phosphorylation, acetylation and so on. These modifications combined with its related proteins, such as reader, writer and eraser, can influence chromatin phase separation and regulate the dynamic of chromatin organization (Figure 1A). Acetylation of core histone and linker H1 has different influences. Acetylation of core histone disrupts the original nucleosome condensation and induces a new phase when mixed with multi-bromodomain proteins [38]. Interestingly, when acetylated and non-acetylated chromatin condensates are close together, they still remain in their respective states and do not fuse, implying that histone modifications may mediate establishment of distinct chromatin compartments. On the other hand, H1K85 acetylation increased H1 binding to core histones, leading to condensed chromatin [44]. Phosphorylation of histone H1 C-terminal IDRs lower the binding affinity of H1 with DNA and reduces partitioning of H1/DNA complexes from bulk to phase-separated droplets [37]. Methylation of histone H3K9 and H3K27 enables condensation of associated binding proteins which are important for heterochromatin formation, and we will discuss this later. It can be seen that LLPS plays an important role in nucleosome-mediated chromatin condensation and remodeling, especially through histone epigenetic modifications [45].Whether other forms of histone modifications, such as ubiquitination and SUMOylation, affect liquid phases needs further study.

### 4.2. Role of LLPS in Chromatin Loop Formation

Chromatin loops bring together distal elements on DNA and have important functions, such as regulation of gene transcription through enhancer–promoter loops. Loops in mammalians are thought to be formed by loop extrusion in which loop-extruding factors, likely SMC complexes, slide along DNA until counter-border elements, such as CTCF [46]. N-terminus of CTCF and 3D geometry of the CTCF–DNA complex act as a roadblock constraining cohesin movement [47]. Recent studies showed that phase separation also plays a role in chromatin loop formation. One classical example is the transcriptional condensates formed near the enhancer and promoter regions which can regulate expression of genes (Figure 1B). High concentrations of transcription factors, cofactors and RNA polymerase II binding at enhancer and promoter sequences bring the transcriptional elements together and form a phase compartment for transcription [48]. Many of these proteins possess IDRs, such as transcription factors (OCT4, ERα, GATA3, and FET protein family members) [49], transcription coregulators (BRD4 and MED1) [50] and transcription elongation factors (DYRK1A) [51]. Studies showed that BRD4 and MED1 can spontaneously form liquid-like condensates in cells, and recruit transcription factors and RNA Pol II [50]. Different transcription factors interact with the mediator to form condensates through its activation domain, and the formation of condensate is related to gene activation [52]. The aggregates of these proteins constitute specific physicochemical microenvironments, which enable efficient gene expression. Super-enhancers composed of a cluster of typical enhancers can recruit unusually high densities of coactivators, which form larger droplets and drive higher levels of transcription of key cell-identity genes. 

Further studies showed that the C-terminal domain (CTD) of RNA polymerase II can undergo phase separation itself, and the number of CTD consensus heptads influence its localization to active transcription foci [53]. In addition, when CTDs are phosphorylated, Pol II are dissolved from the initiation droplets (Figure 1B). Interestingly, phosphorylated Pol II was trapped within novel phase separated droplets that are formed mainly by super elongation complex (SEC). The SEC condensates can concentrate positive transcription elongation factor b (P-TEFb), resulting in paused Pol II release and transcriptional activation [54]. This explains the mechanism by which RNA pol II is recruited to active genes in its unphosphorylated state and released for elongation following phosphorylation of the CTD. These studies showed that LLPS plays a critical role in enhancer–promoter looping interaction and gene expression regulation. Artificially inducing phase condensation at specific genomic loci can pull distal targeted genomic elements together, which support contribution of phase separation to loop formation [55]. In agreement with this, disruption of LLPS weakens enhancer–promoter interactions but has no effect on CTCF-dependent loops [43]. In addition, phase separation of fusion protein in cancer can drive aberrant chromatin looping and ectopic expression of oncogene. Fusion protein resulting from translocation, such as NUP98–HOXA9 recurrently detected in leukemia, contains IDR domain and can induce CTCF-independent DNA looping between enhancers and oncogenes through LLPS, which will then contribute to cancer development [56]. However, some studies showed that depletion of Pol II and mediator or disruption of Pol II-containing condensates elicit little effect on enhancer–promoter interaction, although gene expression was markedly affected [57,58]. Therefore, the relationship between phase separation and loop formation or transcription needs more investigation. 

Recent studies showed that silencers, which are defined by enrichment of H3K27me3 or anchors of PRC2-mediated loops, can also function effectively across long distances through chromatin looping to perform their repressive functions [59,60,61]. Removal of a silencer leads to upregulation of interacting target genes, altered chromatin interactions and abnormal phenotypes. Phase separation-mediated repressor condensation, such as PRC complex, may facilitate these repressed chromatin interactions, similar to how enhancers activate target genes by phase separation of activators. However, this model needs further validation. 

### 4.3. Role of Phase Separation in TAD Assembly and Reprogramming

TADs are also thought to be assembled by loop extrusion and each TAD emerges from multiple loops dynamically formed through extrusion [34,62]. Loop extrusion explains many characters of TADs, such as preferential orientation of CTCF motifs, enrichments of CTCF and cohesin at TAD boundaries and why domains fuse during boundary deletion. However, there are still a lot of experimental discoveries which are hard to explain by loop extrusion. For example, single-cell Hi-C highlights the stochastic nature of TADs, with boundaries varying from cell to cell [63]. In addition, cohesin depletion does not disrupt the TAD structure in single cells [64]. These hint at other mechanisms which may exist for TAD formation. Recent studies illustrate the role of phase separation in TAD establishment and reorganization. 

Theoretical approaches such as polymer physics have been employed to investigate chromatin architecture and unveil the underlying shaping mechanisms [65]. Modeling chromatin as a block copolymer that phase separates through intra-polymer self-interactions recapitulates the characteristic TAD structure, implying the role of phase separation in TAD formation (Figure 1C) [12,66]. Supporting the polymer model, super-resolution electron microscopy can identify ~200- to 300-nm-wide chromatin domains composed of aggregated nucleosomes that overlap with individual TADs [67]. Chromatin-binding proteins contribute to the pattern of polymer–polymer interactions and inclusion of protein binding at target sites increases accuracy of polymer models [3]. TAD stochasticity in single-cell Hi-C data can be recaptured by state degeneracy in polymer models, where population level TADs match the location of the self-assembled globules that more frequently form and thermodynamics degeneracy of phase separation results in variability of TAD-like contact patterns in single cells [68]. These studies imply phase separation underlying the mechanism of TAD formation. In agreement with this, 1,6-HD treatment did not affect CTCF and cohesin abundance on DNA but remarkably decreased TAD insulation [43]. In addition, models combining polymer phase separation and loop extrusion are most consistent with the experimental contact data, further validating phase separation’s contribution to TAD establishment [69]. It is of note that in polymer models, the physical phase separation of polymers may be induced by a different molecular mechanism, such as condensate formation and LLPS, bridging molecules and PPPS, or by direct interaction between epigenetically similar regions [70,71]. 

One recent study illustrates the role of LLPS in TAD reorganization (Figure 1C). Jia et al. showed that TADs are reorganized during somatic cell reprogramming and OCT4 phase separation is critical to this process. Disrupting OCT4 LLPS attenuates TAD reorganization and cell fate reprogramming. This is the first experimental study that directly showed that phase separation of master transcription factor contributes to TAD structure [72]. As there are many other master transcription factors containing IDR, it will be interesting to investigate the relationship of their LLPS with 3D genome and cell fate.

### 4.4. Role of Phase Separation in Nuclear Compartmentalization

A and B compartments of chromatin roughly correspond to the transcribed euchromatin and silenced heterochromatin, respectively. More and more evidence shows that compartmentalization seems to be driven, at least in part, by the phase separation [10,73,74]. As introduced in the part of loop formation, transcriptional activators form condensates near (super)enhancers through LLPS. Similarly, IDRs are present in many repressive chromatin-binding proteins. These repressors form separate phase-separated condensates that incorporate repressive factors and exclude active factors [75]. In such a way, different types of chromatin condensates may drive the separation of A and B compartments. It is of note that A/B compartments are not separated from each other into two large condensates. As the long chromatin can be considered as block copolymers, with alternating blocks consisting of monomers of different types (such as euchromatin and heterochromatin), microphase separation will occur according to polymer theory [76]. This means that clusters of blocks with the same type are relatively small instead of being two large domains formed by different types of blocks. Microphase separation can explain the segregation of euchromatin and heterochromatin. In addition, the alternative localization of different kinds of histone post-modifications along the chromatin also supports the pattern of microphase separation [13,77]. 

LLPS has been validated to contribute to the formation of both constitutive and facultative heterochromatin (Figure 1D). The sign of constitutive heterochromatin is H3K9me. Many studies have revealed the role of LLPS in the formation of constitutive heterochromatin. Heterochromatin protein 1 (HP1), which recognizes and binds H3K9me, can trigger the formation of separated aggregates and provoke DNA/chromatin compaction [78,79]. Oligomerization and phase separation of HP1 contribute to the liquid-like droplets. Phase separation is based on IDRs of HP1 which make multivalent interactions with H3K9me. Phosphorylation of the N-terminal chromatin domain can alter the molecular conformation of HP1 IDRs and stimulate the forming of heterochromatin foci in the nucleus [80]. HP1 condensates further incorporate heterochromatin-associated proteins and exclude general activating transcription factors such as TFIIB (Figure 1D) [75]. Similar to HP1, homologous protein Swi6 in yeast can also combine with H3K9me, leading to the exposure of buried histone residues and increasing multivalent interaction sites, thereby promoting LLPS and heterochromatin formation [79]. In accordance with its role in driving heterochromatin, HP1 is essential for de novo 3D genome organization in early Drosophila embryos [81]. Depletion of HP1a causes decreased interactions between B-compartment regions and leads to reduced segregation of B-compartments. In addition to HP1, histone H1 also undergoes LLPS with DNA or nucleosomes in cells, and is colocalized with heterochromatin, which may contribute to the organization of heterochromatin [42]. 

For the facultative heterochromatin marked by H3K27me, recent studies have shown that it is mediated by the phase separation of Chromobox homolog 2 (CBX2) protein [82]. Polycomb group proteins contribute to development and differentiation through repression of master regulators. CBX2, one component of Polycomb repressive complex 1 (PRC1), can form liquid-like condensate on its own and recruit other components of PRC1 (Figure 1D) [83]. It has been shown that the molecular valency of CBX2 may be changed by the phosphorylation modification of protein, affecting its phase separation [83]. H3K27me marked chromatin regions can be recruited to the CBX2 condensates through an adaptor protein, such as CBX7-PRC1 [84]. The incorporated chromatin is then compacted and transcription is repressed. Similar to HP1 condensates, the condensate formed by CBX2 is not compatible with the puncta formed by H3K27Ac and RNA polymerase II [85]. However, the model of chromatin compaction driven by phase separation was challenged by a recent study which showed that even after CBX2 condensate was dissolved, the chromatin remained in its compacted form, implying that the compaction of chromatin is not the result solely of phase separation [86]. It will be interesting to further clarify the relationship between PRC1 condensates and chromatin compaction. These findings indicate that LLPS is critical for the formation of repressed heterochromatin. For euchromatin, in addition to the transcription active condensates, one recent research work showed that RYBP-mediated phase separation of CTCF also organizes inter-A compartment interactions [87]. In conclusion, compartmentalization of chromatin is driven by independent phase separation systems containing mutually exclusive suppressed and activated chromatin.

## 5. Indirect Role of Phase Separation Compartmentalization on Chromatin Organization

**Figure 2:**
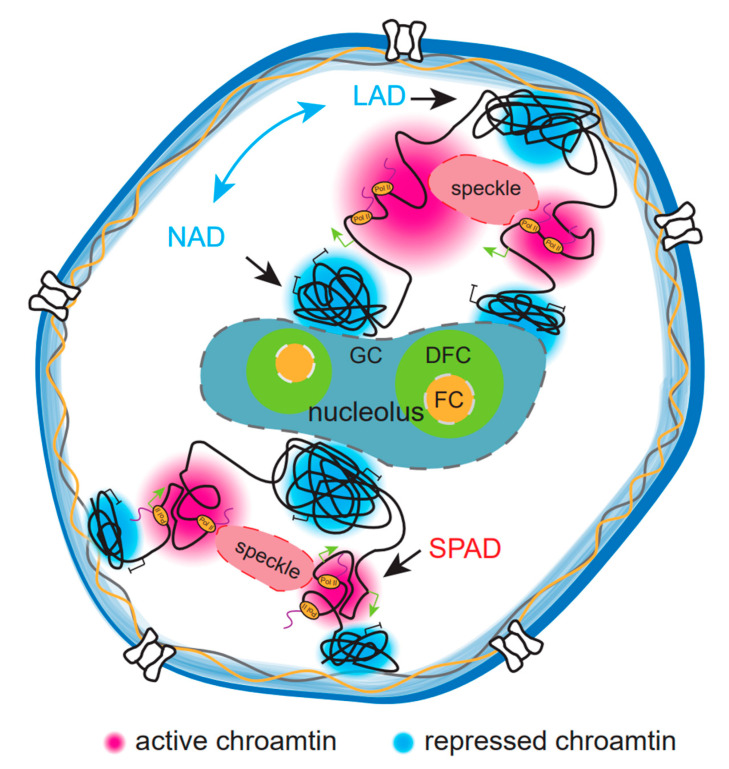
Model for how nuclear sub-compartments shape 3D genome organization. These fixed nuclear elements provide physical limitations for both euchromatin and heterochromatin distribution. As the largest sub-compartment, the nucleolus has three internal phase-separated layers, namely, the fibril center (FC), dense fibrillar component (DFC) and granular component (GC). Transcriptionally inactive heterochromatin clusters (blue) formed by LLPS are widely distributed around nucleolus. Nuclear speckle is also formed by LLPS and is the region for pre-mRNA splicing. Speckle-associated domains (SPADs) tend to be transcriptionally active regions with high concentrations of RNA polymerase II (red). Chromatin associated with nuclear lamina also tend to be condensed heterochromatin. The double arrow indicates the relocation between nucleolar-associated domains (NADs) and lamina-associated domains (LADs) after mitosis.

In addition to the direct participation in formation of various layers of high-order chromatin organization, phase separation can affect overall chromatin structure within the nucleus by influencing interactions between chromatin and nuclear compartmentalization elements, such as nuclear speckles and nucleus. These micron-sized nuclear elements are driven by LLPS and can serve as physical anchors in nucleus. They will restrict chromatin location and mobility in 3D space, and strongly affect overall genome organization. Studies have shown that inactive chromatin accumulates near the nucleolus and nuclear lamina, while nuclear speckles are active chromatin centers (Figure 2). In the following, we will discuss the character of main architectural elements in nuclear and their effects on chromatin structure.

### 5.1. Nuclear Speckles and Chromatin Organization

The nuclear speckles (NSs) are defined as bodies located in the interchromatic region of cell nucleoplasm, which is the region of pre-mRNA splicing and enriched in splicing factors [6]. NSs are found in a wide range of species from plants to animals. There is no substantial difference between the biophysical properties of NSs and nucleoplasm. The density of NSs is slightly higher than that of the surrounding nucleoplasm [88]. Researchers recently found that two proteins, SON and SRRM2, are critical to the formation and organization of NS [89]. With evolution, the IDRs of these two proteins seem to be lengthened, which may promote the weak multivalent interaction required for the formation of this structure [89]. Besides these two main proteins, FISH experiments have demonstrated that polyadenylated RNA and RNA-binding proteins (RBPs) related to pre-mRNA splicing are also enriched in NSs [89,90]. Aggregation of these high concentrations of RBPs which have low-complexity domains may also contribute to the formation of NSs [89]. 

Chromatin regions related to NSs tend to be transcriptionally activated (Figure 2) [91]. Recently, there are a few methods for detecting chromosomal domains associated with nuclear speckle genome-widely, namely, Mapping of RNA Genome Interactions (MARGI), Split-Pool Recognition of Interactions by Tag Extension (SPRITE), Tyramide Signal Amplification (TSA)-seq and Genome Organization using CUT and RUN Technology (GO-CaRT) [29,92,93,94]. These methods detect NS-associated domains (NSADs) by mapping RNA–DNA interaction containing NS-specific RNA (snRNA, 7S RNA, MALAT1) or labeling NSADs using cytological staining. All these methods found that NSADs tend to be active transcriptional regions located in the A compartment and enriched active histone marks. Especially, SPRITE revealed that there are two hubs for interchromosomal interactions, namely, the “active hub” and the “inactive hub”. The active hub was rich in spliceosomal U1 snRNA and Malat1, and was associated with NSs [95]. In agreement with the role of NSs in organizing actively transcribed euchromatin, disruption of NSs by depletion of structural protein SRRM2 reduced spatial chromatin interactions within the A compartment [96]. These results imply that nuclear speckles contribute to chromatin structure mainly through concentrating active chromatin domains. How is an activated chromatin region tethered on NS? A live cell image revealed an actin-dependent, directional movement of plasmid arrays toward nuclear speckles, suggesting that the cytoskeleton may contribute to localization of active chromatin to NS [97]. More studies are needed to specify the mechanism of interaction between NS and active chromatin. 

### 5.2. Nucleolus and Chromatin Organization

The most prominent nuclear sub-structure is the nucleolus, which is a typical membraneless organelle. The nucleolus is the initial site of ribosome biogenesis and involves hundreds of proteins guiding the chemical modification of rRNA and the folding of pre-rRNA. The nucleolus of mammalian cells has three sub-compartments, namely, the fibril center (FC), dense fibrillar component (DFC) and granular component (GC) from inside to outside [98]. Increasing evidence indicates that this layered structure is formed through phase separation [99]. A large number of proteins in nucleolus have IDRs, which are key factors to promote LLPS. For example, the rRNA transcription factors nucleolin and fibrillarin, which are located in DFC, have Gly-Arg-rich domains and can promote phase separation in vitro [100]. Nucleophosmin, which is located in GC, displays many characteristics of phase separation proteins and phosphorylation of its oligomeric domain can regulate the phase separation behavior [101]. Nucleolar methyltransferase TGS1 also contributes to nucleolus coalescence and stability through phase separation [102]. The evidence of saturation concentration provides additional support for an LLPS model in nucleolus [103]. It is worth noting that higher RNA concentration reduces the saturation concentration, that is, phase separation is more likely to occur, implying that RNA also contributes to phase formation of nucleolus [103]. 

The chromatin regions around the nucleolus tend to be repressive heterochromatin (Figure 2) [104]. Previous microscopy-based studies showed that centromeres and telomeres, as well as inactive X chromosome (Xi) of female cells, are usually related to nucleolus in many cell types [105,106,107]. A few methods can be used to identify nucleolar-associated domains (NADs) genome-widely [29]. Nucleoli can be biochemically purified relying on sonication of nuclei, and the DNA of isolated nucleoli was extracted and analyzed [108]. This method illustrates that NADs mainly consist of inactive regions. Recently developed techniques can locate NADs more precisely. SPRITE can detect chromatin regions interacting with nucleolus rRNA transcripts and identify an ‘inactive hub’ closing to the nucleolus [29]. Recently, Cristiana et al. established DNA adenine methyltransferase identification (DamID)-based methodologies taking advantage of engineered nucleolar histone fused with Dam [109]. They also showed that NAD enriched repressive chromatin features and was characterized by higher H3K9me2 and lower H3K27me3 content compared with repressive chromatin in contact with nuclear lamina. The above studies showed that nucleolus can contribute to the chromosome architecture by constraining the localization of heterochromatin regions. It is not clear yet how repressive chromatin regions are tethered on nucleolus. Recent studies suggested that LLPS may play certain roles. Clustering of centromeres and localization to nucleolus periphery in Drosophila are dependent on the nucleophosmin homolog NLP, which can form liquid-like droplets [110]. Treatment of MEF with 1,6-hexanediol which disrupts LLPS reduced the association of NADs with nucleolus [111]. How phase separation helps chromatin domain target nucleolus needs further study.

### 5.3. Nuclear Lamina and Chromatin Organization

The nuclear lamina (NL) is an important nuclear landmark lying in the inner surface of the nuclear membrane and can interact with the chromatin [112]. It is a fibrous network composed of lamin A/C, lamin B1 and lamin B2, and has multiple cellular functions, such as providing physical support for the nucleus, controlling the protein concentration in the nucleus [113]. Although NL was not organized through phase separation, NL has an important effect on chromatin architecture and LLPS may play roles in the interaction of NL with chromatin.

Chromatin related to NL tends to be condensed heterochromatin (Figure 2). Several methods can be used to genome-widely detect lamina-associated domains (LAD) which are in close proximity to the NL, such as (single cell) DamID, GO-CaRT [94,114]. These methods illustrate that LAD is characterized by low gene density, the presence of LINE transposable elements, and enrichment of histone modifications H3K9me and H2K27me, which are all features of repressed genome regions [115,116]. As centers of heterochromatin, both nucleoli and nuclear lamina are positively correlated. NAD and LAD can be relocated to each other after mitosis [117]. However, there is a report that lamins have non-canonical functions to regulate active transcription inside LADs and in-depth research is needed to clarify this [118]. Recent studies showed that NL is important for proper spatial partitioning of repressed chromatin. When lamins are absent, LADs become detached and peripheral genomic regions are relocated to the nuclear interior [119]. Furthermore, active and inactive genomic compartments are intermixed and TAD–TAD interactions are altered. Polymer simulations of retinal rod cells of nocturnal animals, in which heterochromatin is located in the interior of the nucleus, also showed that when interactions between heterochromatin and the lamina are added, the inverted nuclei changed to the conventional organization [120]. 

Phase separation may contribute to NL-chromatin interaction [20,121]. HP1, H3K9me and H3K27me, through which LLPS drives heterochromatin formation, contribute to interaction between NL and chromatin. HP1 can interact with lamin B receptor (LBR) and heterochromatin was sequestered to the periphery [122]. H3K9me and H3K27me are also involved in the establishment and maintenance of NL-chromatin contacts [123]. In addition, in C. elegans, the Histone H3K9 methyl reader CEC-4 has been identified as an anchor between this methylation mark and the NL [124]. Besides heterochromatin localization, phase separation has also been involved in nuclear envelope reformation and function of nuclear pore complexes [125,126]. Considering that many proteins in the nuclear envelope have direct interaction with components of heterochromatin phase, it will be interesting to investigate whether these proteins, such as LBR or CEC-4, participate in the heterochromatin droplets and their influence on chromatin organization. 

Other nuclear bodies formed through liquid–liquid phase separation may also contribute to chromatin structure. Cajal bodies and Promyelocytic Leukemia Nuclear (PML) bodies act as foci to recruit different genes. Cajal bodies associated regions are enriched with transcriptionally active histone genes and U small nuclear or nucleolar RNA (sn/snoRNA) loci [127]. In the vicinity of PML bodies, highly acetylated blocks of chromatin and nascent RNA are accumulated [128]. These nuclear bodies are themselves devoid of DNA but are rounded by chromatin suggesting their role in localization of specific DNA regions and regulation of chromatin organization. Further studies are needed to illustrate this in detail.

## 6. Conclusions and Perspectives

In recent years, study on the function of phase separation within the nucleus is one of the most rapidly developing fields. Phase condensates can attract or repel certain molecules to activate or inhibit various biochemical reaction processes, which is an effective mechanism making the biological process in the crowded nucleus orderly. Current studies showed that LLPS is a key factor in spatial chromatin organization and gene expression regulation. Destroying specific liquid condensate may lead to changes in chromatin organization and the occurrence of various diseases, including cancer. Our review summarizes the latest work on the role of phase separation in 3D chromatin organization from both direct and indirect views. LLPS directly drives 3D chromatin organization from different levels, such as compartments, TADs, loops and nucleosomes condensation. LLPS also indirectly influences 3D chromatin organization by regulating the interaction of chromatin to important nuclear bodies. It is expected to provide certain support for the future study of the underlying mechanism for 3D chromatin organization. In this review, we focus on talking about LLPS. It is worth noting that other kinds of phase separation, such as polymer–polymer phase separation (PPPS) and liquid–gel phase separation (LGPS) may also play roles in chromatin organization [36,70]. In addition, phase separation is not the only mechanism for clustering of genomic regions in vivo [74]. With the rapid progression of the field, more attention is needed to determine whether phase separation really plays a role and to distinguish different kinds of phase separation.

Innovation of experimental techniques will accelerate the study of relevant fields. For example, Shin et al. developed a CRISPR—Cas9-based optogenetic approach (named ‘CasDrop’) which forms phase-separated condensates by artificial induction. Taking advantage of protein binding upon blue light activation, fusion proteins containing IDR were recruited to genomic loci targeted by dCas9, and led to formation of IDR oligomers at the target genomic loci, which would in turn drive localized LLPS. This system gave us a tool to specifically regulate the local biochemical microenvironment of chromatin and is beneficial for the study of the relationship between LLPS and 3D genome [55]. Minglei et al. developed Hi-MS taking advantage of 1,6-HD to dissolve liquid condensation. This method can be used to quantitative measurement of LLPS properties of chromatin-associated proteins and relevant higher-order chromatin structure [129]. For specific proteins, the state of phase separation can be manipulated by mutating key factors and rescuing through IDR fusion [130]. Using these new methods, it will be attractive to illustrate the role of LLPS of specific proteins in chromatin organization and biological processes, such as development and disease. Another interesting topic is determining how cells regulate chromatin organization through altering phase separation behavior to respond rapidly to environmental stimuli. We can predict that there will be more intersections between spatial chromatin genomics and LLPS in the near future. 

In particular, the disease mutations which cause changes in liquid condensation propensity of proteins and in chromatin organizations provide a new direction for the study of the pathogenesis of diseases. Rett syndrome (RTT) is a typical example, which is caused by the mutation of methyl-CpG-binding protein 2 on the X chromosome. These mutants inhibit phase separation at the specific chromatin focus in neurons [131]. Studies also showed that fusion protein in cancer triggers de novo formation of condensates, which recruit and concentrate factors to tumor-specific enhancers, contributing to carcinogenesis. For the diseases related to abnormal phase separation, changing the LLPS state by blocking the multivalent molecular interaction sites with some drugs will be a promising method for the treatment. This is a very attractive research direction, although there is still a lot of basic work to do.

## Data Availability

Not applicable.

## Abbreviations

LLPS: liquid–liquid phase separation; LSPS: liquid–solid phase separation; LGPS: liquid–gel phase separation; PPPS: polymer–polymer phase separation; NPCs: nuclear pore complexes; PML: promyelocytic leukemia; SGs: stress granules; IDRs: intrinsically disordered regions; Hi-C: high-throughput chromosome conformation capture; ChiA-PET: chromatin interaction analysis with paired-end tag; HiChIP: in situ Hi-C followed by chromatin immunoprecipitation; SPRITE: split-pool recognition of interactions by tag extension; GAM: genome architecture mapping; TAD: topological association domain; DBDs: DNA binding domains; ADs: activation domains; CTD: C-terminal domain; SEC: super elongation complex; P-TEFb: positive transcription elongation factor b; HP1: Heterochromatin protein 1; CBX2: Chromobox homolog 2; PRC1: Polycomb repressive complex 1; FC: fibril center; DFC: dense fibrillar component; GC: granular component; SPADs: Speckle-associated domains; NADs: nucleolar-associated domains; LADs: lamina-associated domains; NSs: nuclear speckles; RBPs: RNA-binding proteins; MARGI: Mapping of RNA Genome Interactions; TSA: Tyramide Signal Amplification; GO-CaRT: CUT and RUN Technology; RD-SPRITE: RNA & DNA SPRITE; Xi: inactive X chromosome; NL: nuclear lamina; RTT: Rett syndrome.
